# Biodiversity baseline of the French Guiana spider fauna

**DOI:** 10.1186/2193-1801-2-361

**Published:** 2013-07-30

**Authors:** Vincent Vedel, Christina Rheims, Jérôme Murienne, Antonio Domingos Brescovit

**Affiliations:** Laboratoire d’entomologie Entobios, 5 Bis rue François Thomas, 97310 Kourou, Guyane Française, France; Laboratoire d’écologie intégrative, UMR ECOFOG, Université des Antilles et de la Guyane, Campus Agronomique de Kourou, 97310 Kourou, French Guiana, France; Laboratório Especial de Coleções Zoológicas, Instituto Butantan, Av. Vital Brasil 1500, Butantã, São Paulo, SP Brazil 05503-900; CNRS, EFA, UMR 5174 EDB (Laboratoire Evolution et Diversité Biologique), Université Paul Sabatier, 118 route de Narbonne, F-31062 Toulouse, France

**Keywords:** Araneae, Arachnids, Bio monitoring, French Guiana, Neotropics, Species richness

## Abstract

The need for an updated list of spiders found in French Guiana rose recently due to many upcoming studies planned. In this paper, we list spiders from French Guiana from existing literature (with corrected nomenclature when necessary) and from 2142 spiders sampled in 12 sites for this baseline study. Three hundred and sixty four validated species names of spider were found in the literature and previous authors’ works. Additional sampling, conducted for this study added another 89 identified species and 62 other species with only a genus name for now. The total species of spiders sampled in French Guiana is currently 515. Many other Morphospecies were found but not described as species yet. An accumulation curve was drawn with seven of the sampling sites and shows no plateau yet. Therefore, the number of species inhabiting French Guiana cannot yet be determined. As the very large number of singletons found in the collected materials suggests, the accumulation curve indicates nevertheless that more sampling is necessary to discover the many unknown spider species living in French Guiana, with a focus on specific periods (dry season and wet season) and on specific and poorly studied habitats such as canopy, inselberg and cambrouze (local bamboo monospecific forest).

## Background

Under the Streamline European Biodiversity Inventory 2010 protocols (SEBI) (Butchardt et al. 2010; Jones et al. 2011), species occurrences and abundances are currently only being assessed through survey of birds and butterflies. While there is a general agreement that those groups should continue to be monitored (EEA technical report No 11/2012) (EEA (2005), the Group on Earth Observations Biodiversity Observation Network (GEO BON), the European Biodiversity Observatory Network (EBON) (Reviewed in Scholes et al. 2008) and many authors specialized in this field (De Baan et al. 2012; Cardoso et al. 2011; Feest 2013; Feest et al. 2011) have recommended the survey and monitoring of additional groups to fill the taxonomic and ecological gaps.

Spiders have been identified as a meaningful additional indicator taxon by the European Commission FP7-BioBio project (Targetti et al. 2012) not only because they represent well the local micro-fauna richness, but also because they are easy and cheap to sample, sensitive to changes (Cardoso et al. 2008), have little dispersal potential (New 1999), are abundant and diverse (Foelix 1996), represent differences in other species richness and diversity (Cardoso et al. 2008) and are recognized by stakeholders. Moreover, rigorous sampling protocols have just been set up (Cardoso 2009) and locally adapted (Vedel and Lalagüe 2013). They provide a complementary alternative to Lepidoptera in term of distribution and ecological functions as top predators of soil and lower vegetation communities (Cardoso et al. 2008) and are extremely diverse in tropical rain forest (Sørensen et al. 2002; Pinkus-Rendón et al. 2006; Coddington et al. 2009).

French Guiana is 97% covered by primary forest and hosts an exceptionally diverse and distinctive equatorial forest, part of the Amazonian tropical rainforest. This region also has an increasing demographic and economic development, which will raise conservation issues in the near future. As such it deserves special attention from the scientific community. The last integrative spider species list for French Guiana is almost 70 years old (Di Caporiacco 1954) with few later additions (Drolshagen and Bäckstam 2011; Lopez 1988). In this study, our goal is to establish a baseline biodiversity reference for the spider fauna of French Guiana to enable further studies which will set spider monitoring as an efficient “tool” for assessment and monitoring local biodiversity.

## Results and discussion

2142 spider specimens were sampled and sent for identification during this study. Identification results are summarized in Table 1. About 692 Morphospecies (M-S from hereonin) were singled out from this material with many M-S represented only by singletons. Many of these M-S could not be identified yet, and therefore are not included in the species list. In addition, many individuals could not be identified because they were either juveniles or undescribed, and they were therefore excluded from this list. Individuals identified only at the genus level are mainly species which are either not recognized yet, even with the sexual organs (often just one sex represented), or are a species new to science and therefore not described and not named yet. In any case they represent a species not found in the region until now.

**Table 1:** **Number of specimens collected for each location with the number of morpho-species recognized**

Sampling site	Coordinates	Season	Number of	Number of	Type of	Sampling
			collected individuals	morpho-species	habitats	methods
Crique Baggot	22N0329797-0501628	Wet	24	23	FF	B(1), SN(1), H(1)
Gentry plots (Petite Montagne Tortue, Régina)	22N0362289-0477672	Wet, Dry	97	58	WS, FF, TF	MT, WP
Gentry plots (Laussat Ouest)	22N0213521-0605836	Wet, Dry	76	42	WS, FF, TF	MT, WP
Grand Connétable Island	22N0396505-0534312	Wet	15	10	OI	H(2)
Kaw	22N0315898-0556000	Wet	78	60	WF	B(2), SN(2), S(2)
La Trinité	22N0232748-0510994	Dry	439	242	FF, TF, I	B (6), SN(6), S(3), WP
Mont Itoupé	-	Wet	74	61	I	WP, MT
Nouragues	22N0314321-0446496	Wet	338	175	FF, TF, I	B(6), SN(6), S(3), WP
Nouragues	22N0307547-0450440	Dry	375	270	FF, TF, I	B(10), SN(10), S(5), WP
Piste des compagnons	22N0310766-0564719	Dry	27	25	TF	MT
Saül	22N0253843-0400740	Wet	482	347	FF, TF, I	B(6), SN-6), S(3),
Savane-roche Virginie	22N0257866, W0731672	Dry	117	43	I	SN(2), H(2)
**Total**			**2142**	**NA**		

*Abbreviations*: (*FF* Flooded Forest, *TF* Terra Firme, *I* Inselberg, *WF* Wet Forest, *OI* Oceanic Island, *WS* White Sand, *B* Beating, *SN* Sweep Net, *S* Sieve, *H* by Hand, *WP* Window Pane trap, *MT* Malaise Trap. Numbers in brackets reflect the units of sampling effort for the active techniques applied. The wet season lasts from December until June and the dry season from July until November with some little variations. The total number of M-S cannot be determined (and it is therefore noted *NA*=Not Applicable) because at some sites spiders were not photographed and could not be compared with other sites (those sites are the ones not used for the following analyses).

After adding to Caporiacco (1954) list the sampling from this study and the previously identified materials from the two last authors, we obtained a total number of 515 species belonging to 45 families (Table 2). Therefore, 151 new species were added in this study and nine new families for French Guiana were also found: Amaurobiidae, Cyrtaucheniidae, Hersiliidae, Linyphiidae, Miturgidae, Oonopidae, Prodidomidae, Senoculidae and Synotaxidae (see Table 3 for the detailed list of species). From these 151 new species 89 species were named at the species level (Table 2), which indicates this study added 20% more species names to the French Guianan total. Only 137 species described in Caporiacco’s work (1954), which represent about 40% of the species number, were resampled in our study. This number is probably largely underestimated due to the lack of certain identification for many specimens.

**Table 2:** **Details of number of species from the existing literature added now from the ones found in this study**

Sources	Species level	M-S identified at the
		Genus level
Former list published: (Caporiacco 1954)	364	0
Brescovit’s review (Brescovit et al. 2011)	34	2
Present study’s samples	55	60
Total	453	62

**Table 3:** **Current list of identified spiders from French Guiana with notes about the current names**

Guyane+espèces			
***Familyx***	***Species***	***New finding***	***Notes***
**Amaurobiidae**	*Amaurobius brevis* (Taczanowski, 1874)	B	incertae sedis/ Corinnidae
**Amaurobiidae**	*Amaurobius cayanus* (Taczanowski, 1874)	B	incertae sedis/ Corinnidae
**Amaurobiidae**	*Amaurobius hirtus* (Taczanowski, 1874)	B	incertae sedis/ Corinnidae
**Amaurobiidae**	*Amaurobius rufipes* (Taczanowski, 1874)	B	incertae sedis/ Corinnidae
**Anyphaenidae**	*Aljassa* n. sp.1	V	
**Anyphaenidae**	*Anyphaenoides* n. sp.1	V	
**Anyphaenidae**	*Hibana melloleitaoi* (Caporiacco, 1947)	V	
**Anyphaenidae**	*Katissa* n. sp.1	V	
**Anyphaenidae**	*Mesilla anyphaenoides* (Caporiacco 1954)		
**Anyphaenidae**	*Patrera armata* (Chickering, 1940)	V	
**Anyphaenidae**	*Patrera* n. sp.1	V	
**Anyphaenidae**	*Patrera* n. sp.2	V	
**Anyphaenidae**	*Patrera* n. sp.3	V	
**Anyphaenidae**	*Patrera* n. sp.4	V	
**Anyphaenidae**	*Sillus furciger* (Caporiacco 1954)		
**Anyphaenidae**	*Wulfila* n. sp.1	V	
**Araneidae**	*Actinosoma pentacanthum* (Walckenaer, 1841)		
**Araneidae**	*Acacesia hamata* (Hentz, 1847)		
**Araneidae**	*Acacesia tenella* (Koch, 1871)		
**Araneidae**	*Alpaida deborae* (Levi, 1988)		
**Araneidae**	*Alpaida erythrothorax* (Taczanowski, 1873)		
**Araneidae**	*Alpaida graphica* (Cambridge, 1889)		
**Araneidae**	*Alpaida marmorata* (Taczanowski, 1873)		
**Araneidae**	*Alpaida sulphurea* (Taczanowski, 1873)		
**Araneidae**	*Alpaida truncata* (Keyserling, 1865)		
**Araneidae**	*Alpaida truncata obscura* (Caporiacco 1954,1948)		
**Araneidae**	*Alpaida truncata sexmaculata* (Caporiacco 1954, 1948)		
**Araneidae**	*Alpaida veniliae* (Keyserling, 1865)		
**Araneidae**	*Araneus appendiculatus* (Taczanowski, 1873)		
**Araneidae**	*Araneus contestationis* (Caporiacco 1954)		Nomem dubium
**Araneidae**	*Araneus decaspinus* (Taczanowski, 1873)		Nomem dubium
**Araneidae**	*Araneus guttatus* (Keyserling, 1865)		
**Araneidae**	*Araneus nigrocellatus* (Caporiacco 1954)		Nomen dubium
**Araneidae**	*Araneus venatrix* (Koch, 1838)		
**Araneidae**	*Argiope argentata* (Fabricius, 1775)		
**Araneidae**	*Argiope trifasciata* (Forsskel, 1775)		
**Araneidae**	*Cercidia* n. sp.1		
**Araneidae**	*Chaetacis abrahami* (Mello-Leitão , 1948)	B	
**Araneidae**	*Chaetacis aureola* (Koch, 1836)		
**Araneidae**	*Chaetacis cornuta* (Taczanowski, 1873)		
**Araneidae**	*Chaetacis necopinata* (Chickering, 1960)	V	
**Araneidae**	*Cyclosa fililineata* (Hingston, 1932)	V	
**Araneidae**	*Cyclosa nodosa* (Cambridge, 1889)		
**Araneidae**	*Cyclosa walckenaeri* (Cambridge, 1889)		
**Araneidae**	*Enacrosoma anomalum* (Taczanowski, 1873)		
**Araneidae**	*Epeiroides bahiensis* (Keyserling, 1885)		
**Araneidae**	*Eriophora edax* (Blackwall, 1863)		
**Araneidae**	*Eriophora fuliginea* (Koch, 1838)		
**Araneidae**	*Eriophora nephiloides* (Cambridge, 1889)		
**Araneidae**	*Eustacesia albonotata* (Caporiacco 1954)		
**Araneidae**	*Eustala albicans* (Caporiacco 1954)		
**Araneidae**	*Eustala anastera* (Walckenaer, 1841)		
**Araneidae**	*Eustala clavispina* (Cambridge, 1889)		
**Araneidae**	*Eustala fuscovittata* (Keyserling, 1863)		
**Araneidae**	*Eustala guianensis* (Taczanowski, 1873)		
**Araneidae**	*Eustala lunulifera* (Mello-Leitão, 1939)		
**Araneidae**	*Eustala sagana* (Keyserling, 1893)		
**Araneidae**	*Eustala scutigera* (Cambridge, 1898)		
**Araneidae**	*Eustala semifoliata* (Cambridge, 1899)		
**Araneidae**	*Eustala tridentata* (Koch, 1838)		
**Araneidae**	*Eustala vegeta* (Keyserling, 1865)		
**Araneidae**	*Gasteracantha cancriformis* (Linnaeus, 1758)		
**Araneidae**	*Hingstepeira folisecens* (Hingston, 1932)		
**Araneidae**	*Hypognatha deplanata* (Taczanowski, 1873)		
**Araneidae**	*Hypognatha saut* (Levi, 1996)		
**Araneidae**	*Hypognatha scutata* (Perty, 1833)		
**Araneidae**	*Kapogea sexnotata* (Simon, 1895)		
**Araneidae**	*Mangora melanocephala* (Taczanowski, 1874)		
**Araneidae**	*Mangora saut* (Levi, 2007)	B	
**Araneidae**	*Mangora* n. sp.1	V	
**Araneidae**	*Manogea porracea* (Koch, 1838)		
**Araneidae**	*Metazygia* n. sp.1		
**Araneidae**	*Metepeira brunneiceps* (Caporiacco 1954)		
**Araneidae**	*Metepeira labyrinthea* (Hentz, 1847)		
**Araneidae**	*Micrathena acuta* (Walckenaer, 1841)		
**Araneidae**	*Micrathena gracilis* (Walckenaer, 1805)		
**Araneidae**	*Micrathena cyanospina* (Lucas, 1835)		
**Araneidae**	*Micrathena clypeata* (Walckenaer, 1805)		
**Araneidae**	*Micrathena evansi* (Chickering 1960)		
**Araneidae**	*Micrathena excavata* (Koch, 1836)		
**Araneidae**	*Micrathena fissispina* (Koch, 1836)		
**Araneidae**	*Micrathena flaveola* (Perty, 1839)		
**Araneidae**	*Micrathena hamifera* (Simon, 1897)	B	
**Araneidae**	*Micrathena horrida* (Taczanowski, 1873)		
**Araneidae**	*Micrathena kirbyi* (Perty, 1833)		
**Araneidae**	*Micrathena lata* (Chickering, 1960)		
**Araneidae**	*Micrathena plana* (Koch, 1836)		
**Araneidae**	*Micrathena pungens* (Walckenaer, 1841)		
**Araneidae**	*Micrathena saccata* (Koch, 1836)		
**Araneidae**	*Micrathena schreibersi* (Perty 1833)		
**Araneidae**	*Micrathena sexspinosa* (Hahn, 1822)		
**Araneidae**	*Micrathena spinosa* (Linnaeus, 1758)		
**Araneidae**	*Micrathena triangularis* (Koch, 1836)		
**Araneidae**	*Micrathena triangularispinosa* (De Geer, 1778)		
**Araneidae**	*Micrepeira hoeferi* (Levi, 1995)		
**Araneidae**	*Micrepeira tubulofaciens* (Hingston, 1932)		
**Araneidae**	*Neoscona benjamina* (Walckenaer, 1841)		Nomem dubium
**Araneidae**	*Neoscona nautica* (Koch, 1875)		
**Araneidae**	*Neoscona theisi* (Walckenaer, 1841)		
**Araneidae**	*Ocrepeira albopunctata* (Taczanowski, 1879)		
**Araneidae**	*Ocrepeira covillei* (Levi, 1993)		
**Araneidae**	*Ocrepeira* n. sp.2		
**Araneidae**	*Parawixia audax* (Blackwall, 1863)		
**Araneidae**	*Parawixia kochi* (Taczanowski, 1873)		
**Araneidae**	*Parawixia velutina* (Taczanowski, 1878)		
**Araneidae**	*Scoloderus tuberculifer* (Cambridge, 1889)		
**Araneidae**	*Testudinaria quadripunctata* (Taczanowski, 1879)	B	
**Araneidae**	*Verrucosa arenata* (Walckenaer, 1841)		
**Araneidae**	*Verrucosa septemmammata* (Caporiacco 1954)		
**Araneidae**	*Wagneriana jelskii* (Taczanowski, 1873)		
**Araneidae**	*Wagneriana tayos* (Levi, 1991)	V	
**Araneidae**	*Wagneriana tauricornis* (Cambridge, 1889)		
**Araneidae**	*Wagneriana transitoria* (Koch, 1839)		
**Araneidae**	*Witica cayanus* (Taczanowski, 1873)		
**Araneidae**	*Wixia* n. sp.1		
**Araneidae**	*Xylethrus* n. sp.1		
**Barychelidae**	*Psalistops gasci* (Maréchal, 1996)		
**Caponiidae**	*Nops branicki* (Taczanowski, 1874)		
**Clubionidae**	*Elaver sericea* (Cambridge, 1898)		
**Clubionidae**	*Elaver* n. sp.2	V	
**Corinnidae**	*Apochinomma* n. sp.1	V	
**Corinnidae**	*Castianeira salticina* (Taczanowski, 1874)		
**Corinnidae**	*Castianeira* n. sp.1	V	
**Corinnidae**	*Castianeira* n. sp.2	V	
**Corinnidae**	*Corinna annulipes* (Taczanowski, 1874)		
**Corinnidae**	*Corinna anomala* (Schmidt, 1971)		
**Corinnidae**	*Corinna kochi* (Petrunkevith, 1911)	V	
**Corinnidae**	*Corinna* n. sp.4	V	
**Corinnidae**	*Corinna* n. sp.5	V	
**Corinnidae**	*Corinna* n. sp.6	V	
**Corinnidae**	*Corinna* n. sp.7	V	
**Corinnidae**	*Medmassa septentrionalis* (Caporiacco 1954)		Nomem dubium
**Corinnidae**	*Methesis brevitarsa* (Caporiacco 1954)		
**Corinnidae**	*Myrmecium* n. sp.1	V	
**Corinnidae**	*Myrmecium* n. sp.2	V	
**Corinnidae**	*Myrmecium* n. sp.3	V	
**Corinnidae**	*Myrmecium* n. sp.4	V	
**Corinnidae**	*Myrmecium bifasciatum* (Taczanowski, 1874)		
**Corinnidae**	*Myrmecotypus* n. sp.1	V	
**Corinnidae**	*Parachemmis hassleri* (Gertsch, 1942)	V	
**Corinnidae**	*Parachemmis* n. sp.1	V	
**Corinnidae**	*Paradiestius* n. sp.1	V	
**Corinnidae**	*Tupirinna* n. sp.1	V	
**Corinnidae**	*Trachelas anomalus* (Taczanowski, 1874)		
**Corinnidae**	*Trachelas* n. sp.1	V	
**Corinnidae**	*Trachelas* n. sp.2	V	
**Corinnidae**	*Trachelas* n. sp.3	V	
**Ctenidae**	*Ancylometes bogotensis* (Keyserling, 1876)	B	
**Ctenidae**	*Ancylometes rufus* (Walckenaer, 1837)		
**Ctenidae**	*Centroctenus auberti* (Caporiacco 1954)		
**Ctenidae**	*Ctenus ellacomei* (Cambridge, 1902)		
**Ctenidae**	*Ctenus* n. sp.1	V	
**Ctenidae**	*Ctenus* n. sp.2	V	
**Ctenidae**	*Ctenus* n. sp.3		
**Ctenidae**	*Ctenus villasboasi* (Mello-Leitao, 1949)	V	
**Ctenidae**	*Ctenus crulsi* (Mello-Leitao, 1930)	V	
**Ctenidae**	*Ctenus dubius* (Walckenaer, 1805)		
**Ctenidae**	*Cupiennius bimaculatus* (Taczanowski, 1874)	V	
**Ctenidae**	*Cupiennius foliatus* (Cambridge, 1901)		
**Ctenidae**	*Isoctenus latevittatus* (Caporiacco 1954)		Nomen nudum
**Ctenidae**	*Phoneutria fera* (Perty, 1833)		
**Ctenidae**	*Phoneutria reidyi* (Cambridge, 1897)		
**Cyrtaucheniidae**	*Fufius* n. sp.1	V	
**Deinopidae**	*Deinopis guianensis* (Taczanowski, 1874)		
**Dictynidae**	*Phantyna mandibularis* (Taczanowski, 1874)		
**Dipluridae**	*Harmonicon audeae* (Maréchal & Marty, 1998)		
**Dipluridae**	*Harmonicon oiapoqueae* (Drolshagen & Bäckstam 2011)		
**Dipluridae**	*Harmonicon rufescens* (Cambridge, 1897)		
**Dipluridae**	*Diplura nigra* (Cambridge, 1897)		
**Dipluridae**	*Ischnothele guianensis* (Walkenaer, 1837)		
**Dysderidae**	*Dysdera bicolor* (Taczanowski, 1874)		
**Eresidae**	*Eresus* n. sp.1	V	
**Eresidae**	*Eresus* n. sp.2	V	
**Filistatidae**	*Kukulcania hibernalis* (Hentz, 1842)		
**Gnaphosidae**	*Echemographis distincta* (Caporiacco 1954)		
**Hersiliidae**	*Neotama* n. sp.1	V	
**Hersiliidae**	*Ypypuera* n. sp.1	V	
**Idiopidae**	*Idiops opifex* (Simon, 1889)		
**Linyphiidae**	*Ceratinopsis jelskii* (Keyserling, 1886)	B	Nomem dubium
**Linyphiidae**	*Meioneta* n. sp.1	V	
**Lycosidae**	*Agalenocosa denisi* (Caporiacco, 1947)		
**Lycosidae**	*Aglaoctenus castaneus* (Mello-Leitao, 1942)	V	
**Lycosidae**	*Aglaoctenus guianensis* (Caporiacco 1954)		Nomen dubium
**Lycosidae**	*Trochosa* n. sp.1	V	
**Lycosidae**	*Allocosa* n. sp.1	V	
**Lycosidae**	*Hogna vachoni* (Caporiacco 1954)		
**Lycosidae**	*Hogna ventrilineata* (Caporiacco 1954)		
**Lycosidae**	*Pardosa cayennensis* (Taczanowski, 1874)		
**Mimetidae**	*Ero* n. sp.1	V	
**Mimetidae**	*Ero* n. sp.2	V	
**Mimetidae**	*Gelanor mabelae* (Chickering, 1947)	V	
**Mimetidae**	*Gelanor zonatus* (Koch 1845)		
**Miturgidae**	*Cheiracanthium inclusum* (Hentz, 1847)	B	
**Miturgidae**	*Teminius insularis* (Lucas, 1857)	B	
**Nephilidae**	*Nephila clavipes* (Linnaeus, 1767)		
**Nephilidae**	*Nephila cornuta* (Pallas, 1772)		
**Nephilidae**	*Nephilengys cruentata* (Fabricius, 1775)		
**Nesticidae**	*Nesticus citrinus* (Taczanowski, 1874)		
**Ochyroceratidae**	*Ochyrocera caeruleoamethystina* (Lopez & Lopez, 1997)		
**Oonopidae**	*Neoxyphinus hispidus* (Dumitresco & Georgescu, 1987)	V	
**Oonopidae**	*Oonops* n. sp.1	V	
**Oxyopidae**	*Hamataliwa barroana* (Keyserling 1887)		
**Oxyopidae**	*Oxyopes haemorrhous* (Mello-Leitao, 1929)		
**Oxyopidae**	*Oxyopes maripae* (Caporiacco 1954)		
**Oxyopidae**	*Oxyopes masculinus* (Caporiacco 1954)		
**Oxyopidae**	*Oxyopes salticus* (Hentz, 1845)	V	
**Oxyopidae**	*Peucetia macroglossa* (Mello-Leitão, 1929)		
**Oxyopidae**	*Tapinillus longipes* (Taczanowski, 1872)		
**Paratropididae**	*Paratropis papilligera* (Cambridge, 1896)		
**Philodromidae**	*Cleocnemis punctulata* (Taczanowski, 1872)		
**Philodromidae**	*Philodromus cayanus* (Taczanowski, 1872)		
**Pholcidae**	*Artema atlanta* (Walckenaer, 1837)		
**Pholcidae**	*Litoporus saul* (Huber, 2000)		
**Pholcidae**	*Mesabolivar cyaneus* (Taczanowski, 1874)		
**Pholcidae**	*Metagonia auberti* (Caporiacco 1954)		
**Pholcidae**	*Physocyclus globosus* (Taczanowski, 1874)	B	
**Pholcidae**	*Smeringopus pallidus* (Blackwall, 1858)	B	
**Pisauridae**	*Architis spinipes* (Taczanowski, 1874)		
**Pisauridae**	*Architis tenuis* (Simon, 1898)	B	
**Pisauridae**	*Dolomedes elegans* (Taczanowski, 1874)		
**Pisauridae**	*Thaumasia benoisti* (Caporiacco 1954)		
**Pisauridae**	*Thaumasia marginella* (Koch, 1847)		
**Pisauridae**	*Tinus nigrinus* (Cambridge, 1901)		
**Prodidomidae**	*Zimiris doriai* (Simon, 1882)	B	
**Salticidae**	*Acragas flavescens* (Caporiacco 1954)		Nomen dubium
**Salticidae**	*Albionella guianensis* (Caporiacco 1954)	V	
**Salticidae**	*Alcmena trifasciata* (Caporiacco 1954)		Nomen dubium
**Salticidae**	*Amycus ectipus* (Simon, 1900)	V	
**Salticidae**	*Amycus effeminatus* (Caporiacco 1954)	V	
**Salticidae**	*Amycus favicomis* (Simon, 1900)	V	
**Salticidae**	*Amycus patellaris* (Caporiacco 1954)		Nomen dubium
**Salticidae**	*Anasaitis canosa* (Walckenaer, 1837)		
**Salticidae**	*Beata rustica* (Peckham & Peckham, 1895)		
**Salticidae**	*Breda apicalis* (Simon, 1901)	V	
**Salticidae**	*Capidava variegata* (Caporiacco 1954)		Nomen dubium
**Salticidae**	*Chinoscopus gracilis* (Taczanowski, 1872)		
**Salticidae**	*Chinoscopus maculipes* (Crane, 1943)		
**Salticidae**	*Chira guianensis* (Taczanowski, 1871)		
**Salticidae**	*Chira simoni* (Galiano, 1961)	V	
**Salticidae**	*Chira spinipes* (Taczanowski, 1871)		
**Salticidae**	*Chira thysbe* (Simon, 1902)	V	
**Salticidae**	*Chira trivittata* (Taczanowski, 1871)		
**Salticidae**	*Chirothecia crassipes* (Taczanowski, 1878)		
**Salticidae**	*Chloridusa* n. sp.1	V	
**Salticidae**	*Chloridusa* n. sp.2	V	
**Salticidae**	*Cobanus* n. sp.1	V	
**Salticidae**	*Corythalia heliophanina* (Taczanowski, 1871)		
**Salticidae**	*Corythalia luctuosa* (Caporiacco 1954)		
**Salticidae**	*Corythalia tropica* (Mello-Leitão, 1939)		
**Salticidae**	*Corythalia variegata* (Caporiacco 1954)		Nomen dubium
**Salticidae**	*Corythalia walecki* (Taczanowski, 1871)		
**Salticidae**	*Cyllodania fasciata* (Caporiacco 1954)	V	
**Salticidae**	*Cyllistella* n. sp.1	V	
**Salticidae**	*Dendryphantes coccineocinctus* (Caporiacco 1954)		Nomen dubium
**Salticidae**	*Dendryphantes spinosissimus* (Caporiacco 1954)		Nomen dubium
**Salticidae**	*Euophrys ambigua* (Koch, 1846)		
**Salticidae**	*Eustiromastix bahiensis* (Galiano, 1979)	V	
**Salticidae**	*Eustiromastix guianae* (Caporiacco 1954)		
**Salticidae**	*Eustiromastix major* (Simon, 1902)		
**Salticidae**	*Fluda* n. sp.1	V	
**Salticidae**	*Freya decorata* (Koch, 1846)		
**Salticidae**	*Freya disparipes* (Caporiacco 1954)		
**Salticidae**	*Freya grisea* (Cambridge, 1901)	V	
**Salticidae**	*Freya perelegans* (Simon, 1902)	V	
**Salticidae**	*Frigga coronigera* (Koch, 1846)	V	
**Salticidae**	*Frigga kessleri* (Taczanowski, 1872)		
**Salticidae**	*Habronattus paratus* (Peckham & Peckham, 1896)	V	
**Salticidae**	*Helvetia cancrimana* (Taczanowski, 1872)		
**Salticidae**	*Hypaeus flavipes* (Simon, 1900)	V	
**Salticidae**	*Hypaeus porcatus* (Taczanowski, 1871)		
**Salticidae**	*Hypaeus taczanowskii* (Mello-Leitão, 1948)		
**Salticidae**	*Kalcerrytus* n. sp.1	V	
**Salticidae**	*Kalcerrytus kikkri* (Galiano, 2000)		
**Salticidae**	*Kalcerrytus nauticus* (Galiano, 2000)	V	
**Salticidae**	*Lurio lethierryi* (Taczanowski, 1872)		
**Salticidae**	*Lurio splendidissimus* (Caporiacco 1954)		Nomem dubium
**Salticidae**	*Lurio solennis* (Koch, 1846)		
**Salticidae**	*Lyssomanes amazonicus* (Peckham & Wheeler, 1889)	B	
**Salticidae**	*Lyssomanes elegans* (Cambridge, 1900)		
**Salticidae**	*Lysomanes ipanemae* (Galiano, 1980)		
**Salticidae**	*Lyssomanes longipes* (Taczanowski, 1871)		
**Salticidae**	*Lyssomanes parallelus* (Peckham & Wheeler, 1889)		
**Salticidae**	*Lyssomanes unicolor* (Taczanowski, 1872)		
**Salticidae**	*Maeota dichrura* (Simon, 1901)	V	
**Salticidae**	*Mago acutidens* (Simon, 1900)		
**Salticidae**	*Mago barbatus* (Caporiacco 1954)		Nomen dubium
**Salticidae**	*Mago chickeringi* (Caporiacco 1954)		
**Salticidae**	*Mago dentichelis* (Crane, 1949)		
**Salticidae**	*Mago fasciatus* (Mello-Leitao, 1940)	V	
**Salticidae**	*Mago fulvithorax* (Caporiacco 1954)		Nomen dubium
**Salticidae**	*Mago longidens* (Simon, 1900)		
**Salticidae**	*Mago silvae* (Crane, 1943)		
**Salticidae**	*Menemerus bivittatus* (Dufour, 1831)	V	
**Salticidae**	*Myrmarachne obscura* (Taczanowski, 1872)		Nomen dubium
**Salticidae**	*Nagaina modesta* (Caporiacco 1954)		Nomem dubium
**Salticidae**	*Noegus bidens* (Simon, 1900)		
**Salticidae**	*Noegus fuscimanus* (Simon, 1900)	V	
**Salticidae**	*Noegus petrusewiczi* (Caporiacco 1954)		
**Salticidae**	*Noegus rufus* (Simon, 1900)	V	
**Salticidae**	*Noegus* n. sp.1	V	
**Salticidae**	*Nycerella* n. sp.1	V	
**Salticidae**	*Pachomius dybowskii* (Taczanowski, 1872)		
**Salticidae**	*Parnaenus cyanidens* (Koch, 1846)	V	
**Salticidae**	*Phiale crocea* (Koch, 1846)	V	
**Salticidae**	*Phiale cruentata* (Walckenaer, 1837)		
**Salticidae**	*Phiale rubriceps* (Taczanowski, 1871)		
**Salticidae**	*Phiale septemguttata* (Taczanowski, 1871)		
**Salticidae**	*Phiale gratiosa* (Koch, 1846)		
**Salticidae**	*Phiale guttata* ( Koch, 1846)	V	
**Salticidae**	*Phiale niveoguttata* (Cambridge, 1901)		
**Salticidae**	*Phiale simplicicava* (Cambridge, 1901)	V	
**Salticidae**	*Phiale virgo* (Koch, 184*6*)		
**Salticidae**	*Phidippus guianensis* (Caporiaco, 1947)		
**Salticidae**	*Phidippus triangulifer* (Caporiacco 1954)		Nomen dubium
**Salticidae**	*Platycryptus magnus* (Peckham & Peckham, 1894)	V	
**Salticidae**	*Plexippus paykulli* (Audouin, 1826)	V	
**Salticidae**	*Psecas bubo* (Taczanowski, 1871)		
**Salticidae**	*Pseudopartona ornata* (Caporiacco 1954)		
**Salticidae**	*Rhene jelskii* (Taczanowski, 1871)		
**Salticidae**	*Romitia* n. sp.1	V	
**Salticidae**	*Rudra wagae* (Taczanowski, 1872)		
**Salticidae**	*Salticus albosignatus* (Taczanowski, 1849)		Nomen dubium
**Salticidae**	*Salticus bidens* (Taczanowski, 1872)		Nomen dubium
**Salticidae**	*Salticus cabanisi* (Taczanowski, 1872)		Nomen dubium
**Salticidae**	*Salticus cayanus* (Taczanowski, 1871)		Nomem dubium
**Salticidae**	*Salticus crassipes* (Taczanowski, 1871)		Nomen dubium
**Salticidae**	*Salticus cylindricus* (Walckenaer, 1837)		Nomem dubium
**Salticidae**	*Salticus deplanatus* (Taczanowski, 1871)		Nomem dubium
**Salticidae**	*Salticus dryocopinus* (Taczanowski, 1871)		Nomem dubium
**Salticidae**	*Salticus elaterinus* (Taczanowski, 1871)		Nomem dubium
**Salticidae**	*Salticus emaciatus* (Walckenaer, 1837)		Nomem dubium
**Salticidae**	*Salticus fulvatus* (Fabricius, 1896)		
**Salticidae**	*Salticus hamatinus* (Taczanowski, 1849)		Nomem dubium
**Salticidae**	*Salticus longimanus* (Taczanowski, 1871)		Nomem dubium
**Salticidae**	*Salticus mandibularis* (Taczanowski, 1871)		Nomem dubium
**Salticidae**	*Salticus marmottani* (Taczanowski, 1871)		Nomem dubium
**Salticidae**	*Salticus maronicus* (Taczanowski, 1871)		Nomem dubium
**Salticidae**	*Salticus miniaceus* (Taczanowski, 1871)		Nomem dubium
**Salticidae**	*Salticus minutus* (Taczanowski, 1871)		Nomem dubium
**Salticidae**	*Salticus nigerrimus* (Taczanowski, 1871)		Nomem dubium
**Salticidae**	*Salticus olivacens* (Taczanowski, 1871)		Nomem dubium
**Salticidae**	*Salticus ornatus* (Taczanowski, 1871)		Nomem dubium
**Salticidae**	*Salticus paederinus* (Taczanowski, 1871)		Nomem dubium
**Salticidae**	*Salticus platycephalus* (Taczanowski, 1871)		Nomem dubium
**Salticidae**	*Salticus quadriguttatus* (Taczanowski, 1871)		Nomem dubium
**Salticidae**	*Salticus radoszkowskii* (Taczanowski, 1871)		Nomem dubium
**Salticidae**	*Salticus rubescens* (Walckenaer, 1837)		Nomem dubium
**Salticidae**	*Salticus ruficeps* (Taczanowski, 1871)		Nomem dubium
**Salticidae**	*Salticus salutanus* (Taczanowski, 1871)		Nomem dubium
**Salticidae**	*Salticus sericeus* (Taczanowski, 1871)		Nomem dubium
**Salticidae**	*Salticus sexfasciatus* (Taczanowski, 1871)		Nomem dubium
**Salticidae**	*Salticus simoni* (Taczanowski, 1871)		Nomem dubium
**Salticidae**	*Salticus solskii* (Taczanowski, 1871)		Nomem dubium
**Salticidae**	*Salticus superciliatus* (Walckenaer, 1837)		Nomem dubium
**Salticidae**	*Salticus tenebrosus* (Walckenaer, 1837)		Nomem dubium
**Salticidae**	*Salticus tenuis* (Taczanowski, 1871)		Nomem dubium
**Salticidae**	*Salticus trematus* (Walckenaer, 1837)		Nomem dubium
**Salticidae**	*Salticus tricinctus* (Taczanowski, 1871)		Nomem dubium
**Salticidae**	*Salticus uassanus* (Taczanowski, 1871)		Nomem dubium
**Salticidae**	*Salticus verrauxi* (Taczanowski, 1871)		Nomem dubium
**Salticidae**	*Sarinda atrata* (Taczanowski, 1871)		
**Salticidae**	*Sarinda cayennensis* (Taczanowski, 1871)		
**Salticidae**	*Sarinda longula* (Taczanowski, 1871)		
**Salticidae**	*Scopocira melanops* (Taczanowski, 1871)		
**Salticidae**	*Siloca septentrionalis* (Caporiacco 1954)		
**Salticidae**	*Soesilarishius* n. sp.1	V	
**Salticidae**	*Soesilarishius* n. sp.2	V	
**Salticidae**	*Synemosyna myrmeciaeformis* (Taczanowski, 1871)		
**Salticidae**	*Synemosyna subtilis* (Taczanowski, 1871)		Nomem dubium
**Salticidae**	*Synemosyna lucasi* (Taczanowski, 1871)		
**Salticidae**	*Thiodina branicki* (Taczanowski, 1871)		
**Salticidae**	*Thiodina melanogaster* (Mello-Leitão, 1917)	V	
**Salticidae**	*Thiodina pallida* (Koch, 1846)		
**Salticidae**	*Tutelina iridea* (Caporiacco 1954)		Nomem dubium
**Salticidae**	*Viciria chabanaudi* (Fage, 1923)		
**Salticidae**	*Wedoquella* n. sp.1		
**Salticidae**	*Zuniga magna* (Peckham & Peckham, 1892)	B	
**Scytodidae**	*Scytodes fusca* (Walckenaer, 1837)		
**Scytodidae**	*Scytodes lineatipes* (Taczanowski, 1874)		
**Scytodidae**	*Scytodes longipes* (Lucas, 1844)		
**Senoculidae**	*Senoculus canaliculatus* (Cambridge, 1902)	B	
**Senoculidae**	*Senoculus maronicus* (Taczanowski, 1872)		
**Sparassidae**	*Guadana* n. sp.1	V	
**Sparassidae**	*Olios cayanus* (Taczanowski, 1872)		
**Sparassidae**	*Olios nigriventris* (Taczanowski, 1872)		
**Sparassidae**	*Olios quinquelineatus* (Taczanowski, 1872)		
**Sparassidae**	*Olios roeweri* (Caporiacco 1954)		
**Sparassidae**	*Olios rubripes* (Taczanowski, 1872)		
**Sparassidae**	*Olios velox* (Simon, 1880)	V	
**Sparassidae**	*Polybetes pythagoricus* (Holmberg, 1875)		
**Sparassidae**	*Pseudosparianthis megalopalpa* (Caporiacco 1954)		
**Sparassidae**	*Sampaiosia crulsi* (Mello-Leitão, 1930)	V	
**Sparassidae**	*Sparianthina rufescens* (Mello-Leitão, 1940)	V	
**Sparassidae**	*Sparianthis amazonica* (Simon, 1880)	V	
**Sparassidae**	*Thomasettia* n. sp.1	V	
**Sparassidae**	*Vindullus gracilipes* (Taczanowski, 1872)		
**Synotaxidae**	*Synotaxus* n. sp.1	V	
**Tetragnathidae**	*Azilia vachoni* (Caporiacco 1954)		
**Tetragnathidae**	*Chrysometa minuta* (Keyserling, 1883)		
**Tetragnathidae**	*Leucauge acuminata* (Cambridge, 1889)		
**Tetragnathidae**	*Leucauge argyra* (Walckenaer, 1849)		
**Tetragnathidae**	*Leucauge branickii* (Taczanowski, 1874)		
**Tetragnathidae**	*Leucauge funebris* (Mello-Leitão, 1930)		
**Tetragnathidae**	*Leucauge pulcherrima* (Keyserling, 1865)		
**Tetragnathidae**	*Leucauge saphes* (Chamberlain & Ivie, 1936)		
**Tetragnathidae**	*Leucauge taczanowskii* (Marx, 1893)		
**Tetragnathidae**	*Leucauge venusta* (Walckenaer, 1841)		
**Tetragnathidae**	*Metabus ocellatus* (Keyserling, 1864)	B	
**Tetragnathidae**	*Opas caudacuta* (Taczanowski, 1873)		
**Tetragnathidae**	*Opas lugens*, (Cambridge 1896)		
**Tetragnathidae**	*Tetragnatha filiformata* (Roewer, 1942)		
**Tetragnathidae**	*Tetragnatha gibbula* (Roewer, 1942)		
**Theraphosidae**	*Acanthopelma beccarii* (Caporiacco 1954)		
**Theraphosidae**	*Acanthoscurria simoensi* (Vol, 2000)		
**Theraphosidae**	*Avicularia avicularia* (Linnaeus, 1758)		
**Theraphosidae**	*Avicularia avicularia variegata* (Cambridge, 1896)		
**Theraphosidae**	*Avicularia metallica* (Ausserer, 1875)		
**Theraphosidae**	*Avicularia holmbergi* (Thorell, 1890)		
**Theraphosidae**	*Avicularia lycosiformis* (Koch, 1846)		Nomen dubium
**Theraphosidae**	*Avicularia surinamensis* (Strand, 1907)		
**Theraphosidae**	*Ephebopus cyanognathus* (West & Marshall, 2000)		
**Theraphosidae**	*Ephebopus murinus* (Walckenaer, 1837)		
**Theraphosidae**	*Ephebopus rufescens* (West & Marshall, 2000)		
**Theraphosidae**	*Hapalopus guianensis* (Caporiacco 1954)		
**Theraphosidae**	*Magulla Janeira* (Keyserling, 1891)		
**Theraphosidae**	*Neostenotarsus* (Tesmoingt & Schmidt, 2002)		
**Theraphosidae**	*Tapinauchenius gigas* (Caporiacco 1954)		
**Theraphosidae**	*Tapinauchenius violaceus* (Mello-Leitão, 1930)		
**Theraphosidae**	*Teraphosa blondi* (Latreille, 1804)		
**Theraphosidae**	*Vitalius vellutinus* (Mello-Leitao, 1923)		
**Theridiidae**	*Achaearanea hieroglyphica* (Mello-Leitão, 1940)		
**Theridiidae**	*Anelosimus chickeringi* (Levi, 1956)	B	
**Theridiidae**	*Anelosimus eximius* (Keyserling, 1884)	B	
**Theridiidae**	*Anelosimus jucundus* (Cambridge, 1896)	B	
**Theridiidae**	*Anelosimus nigrescens* (Keyserling 1884)	B	
**Theridiidae**	*Anelosimus rupununi* (Levi, 1956)	B	
**Theridiidae**	*Anelosimus studiosus* (Hentz, 1850)	B	
**Theridiidae**	*Argyrodes benedicti* (Lopez 1988)		
**Theridiidae**	*Argyrodes coactatus* (Lopez 1988)		
**Theridiidae**	*Argyrodes elevatus* (Taczanowski, 1873)		
**Theridiidae**	*Argyrodes nephilae* (*Taczanowski*, *1873*)		
**Theridiidae**	*Chrysso albomaculata* (Cambridge, 1882)		
**Theridiidae**	*Chrysso pulcherrima* (Mello-Leitão, 1917)	B	
**Theridiidae**	*Coleosoma acutiventer* (Keyserling, 1884)	B	
**Theridiidae**	*Cryptachaea hirta* (Taczanowski, 1873)		
**Theridiidae**	*Cryptachaea migrans* (Keyserling, 1884)		
**Theridiidae**	*Cryptachaea pusillana* (Roewer, 1942)		
**Theridiidae**	*Cryptachaea rostrata* (Cambridge, 1864)		
**Theridiidae**	*Dipoena* n. sp.1	V	
**Theridiidae**	*Dipoena* n. sp.2	V	
**Theridiidae**	*Episinus* n. sp.1		
**Theridiidae**	*Faiditus americanus* (Taczanowski, 1874)		
**Theridiidae**	*Faiditus caudatus* (Taczanowski, 1874)		
**Theridiidae**	*Faiditus dracus* (Chamberlin & Ivie, 1936)	B	
**Theridiidae**	*Faiditus globosus* (Keyserling, 1884)	B	
**Theridiidae**	*Neospintharus triangularis* (Taczanowski, 1873)	B	
**Theridiidae**	*Parasteatoda tepidariorum* (Koch, 1841)	B	
**Theridiidae**	*Rhomphaea paradoxa* (Taczanowski, 1896)	B	
**Theridiidae**	*Steatoda ancorata* (Holmberg, 1876)	B	
**Theridiidae**	*Nesticodes rufipes* (Lucas, 1846)		
**Theridiidae**	*Theridion incertissimum* (Caporiacco 1954)		
**Theridiidae**	*Theridion rubrolineatum* (Taczanowski, 1874)		Nomem dubium
**Theridiidae**	*Theridula gonygaster* (Simon, 1873)	B	
**Theridiosomatidae**	*Naatlo splendida* (Taczanowski, 1879)	B	
**Theridiosomatidae**	*Plato juberthiei* (Lopez, 1996)		
**Thomisidae**	*Acentroscelus guianensis* (Taczanowski, 1872)		
**Thomisidae**	*Acentroscelus nigrianus* (Mello-Leitão, 1929)		
**Thomisidae**	*Acentroscelus* n. sp.1	V	
**Thomisidae**	*Bucranium taurifrons* (Cambridge, 1881)		
**Thomisidae**	*Bucranium* n. sp.1	V	
**Thomisidae**	*Diaea* n. sp.1	V	
**Thomisidae**	*Epicadus heterogaster* (Guérin, 1829)		
**Thomisidae**	*Epicadinus trispinosus* (Taczanowski, 1872)		
**Thomisidae**	*Erissus truncatifrons* (Simon, 1895)		
**Thomisidae**	*Misumena citreoides* (Taczanowski, 1872)		
**Thomisidae**	*Misumena maronica* (Caporiacco 1954)		
**Thomisidae**	*Misumena nigripes* (Taczanowski, 1872)		
**Thomisidae**	*Misumenops guianensis* (Taczanowski, 1872)		
**Thomisidae**	*Monaeses lucasi* (Taczanowski, 1872)	V	
**Thomisidae**	*Onoculus echinatus* (Taczanowski, 1872)		
**Thomisidae**	*Onoculus pentagonus* (Keyserling, 1880)	V	
**Thomisidae**	*Platyarachne episcopalis* (Taczanowski, 1872)		
**Thomisidae**	*Runcinioides argenteus* (Mello-Leitão, 1929)		
**Thomisidae**	*Stephanopis quinquetuberculata* (Taczanowski, 1872)		
**Thomisidae**	*Strophius* n. sp.1	V	
**Thomisidae**	*Synema aequinoctiale* (Taczanowski, 1872)		
**Thomisidae**	*Synema bipunctatum* (Taczanowski, 1872)		
**Thomisidae**	*Synema bishopi* (Caporiacco 1954)		
**Thomisidae**	*Synema maculatovittatum* (Caporiacco 1954)		
**Thomisidae**	*Tmarus candefactus* (Caporiacco 1954)		
**Thomisidae**	*Tmarus geayi* (Caporiacco 1954)		
**Thomisidae**	*Tmarus grandis* (Mello-Leitão, 1929)		
**Thomisidae**	*Tmarus hystrix* (Caporiacco 1954)		
**Thomisidae**	*Tmarus intentus* (Cambridge, 1892)		
**Thomisidae**	*Tmarus jelskii* (Taczanowski, 1872)		
**Thomisidae**	*Tmarus littoralis* (Keyserling, 1880)		
**Thomisidae**	*Tmarus obesus* (Mello-Leitão, 1929)		
**Thomisidae**	*Tobias albovittatus* (Caporiacco 1954)		
**Thomisidae**	*Tobias corticatus* (Mello-Leitão, 1917)		
**Thomisidae**	*Tobias cornutus* (Taczanowski, 1872)		
**Thomisidae**	*Tobias taczanowskii* (Roewer, 1951)	V	
**Thomisidae**	*Tobias trituberculatus* (Taczanowski, 1872)		
**Thomisidae**	*Uraarachne vittata* (Caporiacco 1954)		
**Titanoecidae**	*Goeldia patellaris* (Simon, 1892)		
**Trechaleidae**	*Enna jullieni* (Simon & Duss, 1898)		
**Trechaleidae**	*Paradossenus longipes* (Taczanowski, 1874)		
**Trechaleidae**	*Rhoicinus* n. sp.1	V	
**Trechaleidae**	*Syntrechalea reimoseri* (Caporiacco, 1947)	V	
**Trechaleidae**	*Trechalea* n. sp.1	V	
**Trechaleidae**	*Trechalea* n. sp.2	V	
**Trechaleidae**	*Trechalea* n. sp.3	V	
**Uloboridae**	*Miagrammopes* n. sp.1	V	
**Uloboridae**	*Philiponnella semiplumosa* (Simon, 1893)		
**Uloboridae**	*Zosis geniculata* (Olivier, 1789)		
**Zodariidae**	*Tristichops coerulescens* (Taczanowski, 1874)		Nomem dubium

The numbers assigned to the M-S indicate only the order they were examined. B means that this species was not taken into consideration by the former list but was found published in the literature by Brescovit et al. 2011 and therefore added. V means that this species is new for French Guiana and found during the sampling expeditions organized for this study.

The number of M-S found (692 M-S for 1617 spiders sampled) is similar to what was found in Bolivia and Peru; respectively 329 species out of 1109 specimens sampled and 635 species for 1821 specimens (reviewed in Coddington et al. 2009), but represents ten times more than the number of spider species found in temperate forests (Coddington et al. 1996) and much more than found in Tanzania (170 species for 9096 specimens sampled) (Sørensen et al. 2002) and Malaysia (578 species for 6999 specimens collected) (Floren and Deeleman-Reinhold 2005) indicating the high diversity of the Amazonian areas. In Guyana, a neighbourhood country of French Guiana, only 351 species were found out of 5965 specimens collected (Coddington et al. 2009). Nonetheless, these comparisons have to be made with caution due to differences in sampling protocols including different techniques, efforts and the number of different habitats studied.

It is interesting to observe that the most diverse families are representative of most of the main feeding guilds of spiders (Dias et al. 2010; Cardoso et al. 2011): Salticidae with 153 species belong to the diurnal hunting spiders, the Araneidae with 113 species belong to the orb weavers, the Thomisidae with 39 identified species are representative of the ambush spiders, the Theridiidae with 33 species represent the entangled web weavers, the Corinnidae with 26 recognized species belong to the nocturnal hunting spiders and finally the Theraphosidae (the most diverse and numerous Mygalomorphae representative) with 17 species, ambush from their burrows. Nevertheless, the Ctenidae (15 species) and Trechaleidae (7 species) are probably much more diverse than we have found up until now. In this study, we collected about 60 different M-S belonging to the first family and 20 for the second.

The rate of Endemism is quite high, as noted by Caporiacco (1954) with 192 species out of 357 (53.8%) never having been found outside French Guiana. In this study we cannot accurately evaluate endemism because of the bias made during the identification: most of the named species are fully identified because they were previously discovered and described from somewhere else, where spider studies were performed over a longer period of time. In any case, the number of endemic species has to be handled carefully here due to the poor comparison possible due to few studies achieved in the neighbouring regions (Venezuela, Surinam, Guyana and Amapá and Pará States in Brazil).

**Figure 1:**
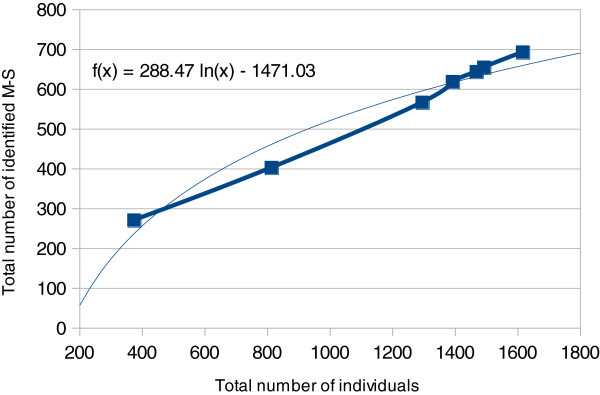
**Accumulation curve showing the slope of the increase of M-S numbers by the number of specimens collected.** The tendency curve (light blue) was added, including its equation.

The accumulation curve shows a constant increase of the number of M-S (Table 4 and Figure 1) and does not reach a plateau, even on the calculated tendency curve. This plateau normally shows the total number of species present in a site or a region. Here, we cannot determine yet this number from the curve which indicates that we have to sample many more individuals to arrive at this plateau.

**Table 4:** **Sampling sites used in the accumulation curve**

Sites	Number of individuals	Number of M-S	Number of « new » M-S in each site
Nouragues (dry season)	375	270	270
Crique Baggot	24	23	11
La Trinité	439	242	132
Gentry plots (Laussat Ouest	76	42	25
Gentry plots (Petite Montagne Tortue, Régina)	97	58	52
Saül	482	347	164
Savane roche Virginie	124	50	38
**Total**	**1617**		**692**

Sites were written in the chronological order they were sampled. The fourth column indicates the number of M-S not found in the previous sampled sites, starting from the top. Therefore they are considered as “new” for the sampling.

From the estimators, the minimum richness is evaluated at 1241 species by the *Chao 1* estimator and the Jackknife estimator calculated 1680 (+/−112) species. These results suggest we only know about one third (515 species identified out of around 1,500 species estimated) of the local spider fauna, which places French Guiana as a region of high diversity of spiders but also in a region where sampling and identification efforts have to be substantially increased to gain a sufficient knowledge in order to be able to use spider as a biodiversity assessment tool.

Now, although the aim of having a list of spiders to start with is reached, those results have to be taken as a first step in the arachnological biodiversity assessment of French Guiana and not as an exhaustive catalog of the spiders inhabiting the area. At each sampled site, although an inclusive sampling protocol was applied, the large number of singletons (between 50 to 70% of the individuals) indicates clearly that those sites are under-sampled (Coddington et al. 2009). A sampling effort index comparing the ratios of abundance to species (N/S) (Colwell and Coddington 1994) gives a result of 54 (9096/170) for Tanzania, 12 for Malaysia, 17 for Guyana, 2.87 for Pérù and 3.37 for Bolivia. In our study, the ratio is 2.34 (1617/692) which shows, in comparison to the other studies both that French Guianan sites are under-sampled and that the diversity is locally high; being comparable to Bolivia and Perù. Tanzania’s study exhibits a high ratio because the sampling was intensive and also because the sites there were poorer in term of diversity.

Moreover, each site should be sampled at different times of the year as the wet and the dry seasons exhibit radical changes in environmental conditions which trigger a change in the communities of animals. In addition, some specific habitats such as canopies, inselbergs (granitic hill specific vegetation) or liana forests were not well sampled and might hold some unknown spider species too. Therefore, in order to assess the total species richness of the spiders of French Guiana, the sampling effort has to be substantially increased, in order that further quantitative studies applying more advanced methods to define the local fauna (Feest & Cardoso 2012). Applying rigorous sampling protocols can be widely applied for local biodiversity assessment using spiders.

## Conclusions

This study sets a starting point for the spider richness described and expected for French Guiana, in order to be able to use spiders as a “bioindication tool”, as recommended by several commissions and organizations, for future biodiversity assessments.

After a literature review and several sampling excursions, the total number of spider species found in French Guiana is now at 515. This revision added 151 new species and nine new families for this region, which make a more credible start for upcoming spider studies. Many other M-S were found but not described as species yet. This study shows that few collections in Guianese forestry habitats brought many new species to the list for French Guiana, which suggests both that the area holds a high diversity of spiders and that this diversity was poorly explored.

The accumulation curve does not yet show the maximum of species number inhabiting this equatorial region but the species richness indices shows the tropical rainforest of French Guiana would host between 1241 and 1792 spider species. They also indicate that more sampling is necessary to complement our current knowledge in this regard. Finally, we can speculate that sampling specific periods (dry season and wet season) and specific and poorly studied habitats such as canopy, inselberg, cambrouze would bring many unknown spider species.

## Methods

### Published data compilation

The official National Natural Patrimony Inventory (INPN: http://inpn.mnhn.fr) currently lists 138 Araneae species for French Guiana. This list was checked and augmented by a complete survey of the literature from a database of Neotropical spiders (Brescovit et al. 2011). Species names were checked with reference to the world catalog of spiders 13.0 (Platnick 2012) and Prószyński’s (2012) catalog of world.

### Study sites

**Figure 2:**
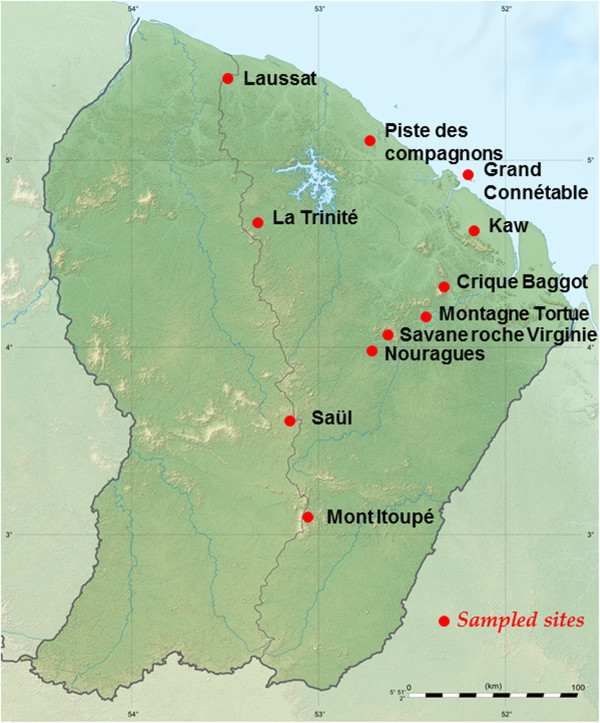
**Map of French Guiana with the sampling sites.**

Sampling was performed by the authors in 12 sites of forest in French Guiana (Figure 2) at different periods during 3 years (2009–2011), to complement the number of species found in the existing literature. The name of the sites are given to the forest area were samples were collected. All of these sites are undisturbed forestry habitats. These sites were chosen for insect collecting expeditions organized by INRA (Institut National de la Recherche Agronomique) (the two Gentry sites), by the CNRS (Kaw) by the first author (Crique Baggot, Savane Roche Virginie, Piste des compagnons), and the remainder by the SEAG (Société Entomologique des Antilles et de la Guyane) for all the other sites.

The sites are located in different part of French Guiana covering almost all the region except the extreme southeast and the west (Figure 2), where most samples of Caporiacco’s study were conducted (Caporiacco 1954). At each site several habitats (such as river side, swamps and terra firme) were sampled (Table 1 for details), offering altogether a good geographical coverage of the spiders found in French Guiana.

### Sampling protocol

In order to collect the maximum number of spiders from every ecological stratum of each habitat, different active techniques and innovative methods such as traps were used (Vedel et al. 2011). Active techniques consist of sampling spiders using a sieve for filtering the soil and the leaf litter, a triangular sweep net for catching spiders inhabiting the low vegetation from 10 cm to 1.5 m, a beating tray to collect individuals living from 1.5 m to 2.5 m in the higher vegetation and by hand to collect visible spiders on trunk or on silk webs. The innovative methods were Malaise traps and window pane traps, normally used to catch insects, which were recognized as efficient at collecting spiders (Vedel et al. 2011). Because of the heterogeneity of the sampling and the initial aim of the study, which was only to increase the number of spider species found, rigorous standardized protocol (Vedel and Lalagüe 2013) was applied only at only the sites Nouragues (wet season), Nouragues (dry season), Crique Baggot, La Trinité, Saül and Savane Roche Virginie. The sampling effort is an equal mix of the active techniques described above where one unit represents one hour of an active sampling technique. For the two Gentry plots spiders were collected only by traps (six window pane traps and six Malaise traps on each site) over a six months period (dry season). At the Mont Itoupe site, six window pane traps and six Malaise traps over one month (dry season) were used to collect spiders. At the Piste des compagnons site, two Malaise traps were placed over a two month period (dry season). Therefore, because of this non-standardization of sampling effort and methods, only a global estimation of total number of species was statistically feasible and no advanced biodiversity studies on spiders (Feest & Cardoso 2012; Cardoso et al. 2009) were possible.

### Storage and identification

Spiders were stored in labeled tubes containing 70% ethanol. Material was identified first as Morphospecies (M-S) by the authors and when possible, identified at the species level by them. Otherwise, material was sent to family specialists (see Acknowledgments) for a complete identification or description. Juveniles were excluded from the list. Specimens are noted as “sp. n” only when recognized as a species new to science by a specialist of the family who will describe it in a further publication.

### Data analysis

An accumulation curve plotting the number of collected individuals by the number of M-S found was drawn to assess the species richness found in French Guiana. This accumulation curve was drawn with only seven of the sampling sites, because a quantitative protocol accompanied with photography of each specimen were applied only at those sites (Vedel and Lalagüe 2013). Although the number of sites (seven) for any statistical study is low, a rough estimation of the total number of spider species found can be nevertheless computed with a high standard deviation to consider. A logarithmic tendency curve (Colwell and Coddington 1994) (and its equation) was also drawn by Excel open office to derive the rate of the M-S number increase from our samplings (Table 4 and Figure 1).

In addition, to estimate the total species richness of French Guiana we computed, from our sampling, the two most widely used estimators: the *Chao 1* (Chao 2005, Gotelli and Colwell 2010) was manually calculated, and the *Jackknife* computed online (http://www.mbr-pwrc.usgs.gov/software/specrich.html) (Burnham and Overton 1979). *Chao 1* is a minimum estimator of the species richness particularly adapted when the number of singletons and doubletons are high, and therefore well adapted to our case. *Jackknife* allows a non-biased estimation of the richness which is complementary of the first estimator used.
